# Immunohistochemical Evaluation and Clinicopathological Correlation of Mer and Axl Tyrosine Kinase TAM Receptors in Cutaneous Melanoma

**DOI:** 10.5826/dpc.1002a29

**Published:** 2020-04-03

**Authors:** Andrea Pontara, Giovanni Paolino, Vanesa Gregorc, Santo Raffaele Mercuri, Alessandra Bulotta, Pietro Bearzi, Claudio Doglioni, Nathalie Rizzo

**Affiliations:** 1 Internal Medicine, IRCCS San Raffaele Scientific Institute, Milan, Italy; 2 Unit of Dermatology, IRCCS San Raffaele Scientific Institute, Milan, Italy; 3 Department of Internal Medicine and Medical Specialties, Dermatology Clinic, La Sapienza-University of Rome, Italy; 4 Department of Medical Oncology, IRCCS, San Raffaele Scientific Institute, Milan, Italy; 5 Department of Pathology, IRCCS San Raffaele Scientific Institute, Milan, Italy

**Keywords:** malignant melanoma, tyrosine kinase, Mer, Axl, receptors, therapy, TAM

## Abstract

**Background:**

Malignant melanoma (MM) is potentially the most dangerous form of skin tumor. In the last few years, the so-called TAM receptors, a unique family of tyrosine kinase (TK) receptors, have become increasingly important.

**Objectives:**

To evaluate Mer and Axl TAM receptor expression to find clinicopathological features that could explain the biological behavior of MM.

**Patients and Methods:**

Clinicopathological data were obtained from an MM electronic database at our Institute. We reviewed 24 cutaneous MM specimens. TAM receptor expression was assayed using immunohistochemistry. Combinative semiquantitative scoring was used for the evaluation of TAM receptor expression (MerTK and AxlTK). Appropriate statistical methods were used to evaluate a possible correlation between TAM receptor expression and the clinicopathological variables of the MM samples (univariate analysis and multivariate analysis).

**Results:**

MerTK and AxlTK were expressed differently in the MM samples, with a major expression of the first receptor. The cells of the tumor microenvironment contributed to the majority of the total score. A significant association was found between AxlScore and the site of the tumor and between AxlScore and the variable ulceration; another correlation was found between MerScore and the following characteristics: pathological stage of the tumor (pT), sex, ulceration, and tumor-infiltrating lymphocytes.

**Conclusions:**

All correlations between the expression of MerTK and AxlTK with the clinical and histological variables of MM should be validated in a large group of people in order to increase the validity and the impact of our observations, with subsequently therapeutic implications in the era of the “targeted therapy.”

## Introduction

Malignant melanoma (MM) is potentially the most dangerous form of skin tumor, causing 90% of skin cancer mortality [1]. Intermittent sun exposure and sunburns are strongly related to the development of MM; however, other factors may be involved in its pathogenesis [2].

MM has more mutations than any other cancer type, and genome aberrations are present in the majority of them. In this regard, oncogenic mutations in c-KIT, NRAS, and BRAF components of the MAPK pathway have been identified in nearly 90% of cutaneous MM [3]; in particular, BRAF and NRAS mutations are the most frequently observed [4].

Regarding treatments, highly selective BRAF and MEK inhibitors have demonstrated impressive clinical results [5]. However, the short duration of response, the acquired resistance in most cases, and the toxicity issues support the rationale for drug combination approaches to improve the outcome of MAPK inhibitors, increasing their efficacy and preventing and/or overcoming resistance [6].

In the last few years, the so-called TAM receptors (Mer, Axl, and Tyro3), a unique family of tyrosine kinase (TK) receptors, have become increasingly important, with a potential role in the era of targeted therapy [7]. Specifically, Axl shows a role in the regulation of invasion and motility of tumoral cells; Tyro3 promotes tumor proliferation and acts as a positive regulator of MITF in MM, while Mer promotes cellular proliferation rather than migration [7–9]. However, their specific role in MM is still unknown, given the lack of scientific articles addressing the subject. For these reasons we aimed to conduct an exploratory evaluation of Mer and Axl TAM receptor in primary MM and to find clinicopathological features that could be associated with their expression.

## Materials and Methods

Clinical and pathological data were obtained from an MM electronic database at our Institute, selecting a period of research between January 1, 2010, and December 31, 2017.

We reviewed 24 MM specimens, divided by sex (male or female), age (≤60 or >60 years), anatomical site (axial [head-neck, thorax-thoracic dorsum, abdomen] or peripheral [upper and lower limbs]), Breslow thickness (≤0.8 or ≥0.81), mitotic rate (<1/mm^2^ or ≥1/mm^2^), ulceration, tumor-infiltrating lymphocytes (TIL), pigmentation, regression, peritumoral vascular invasion, nevus-associated MM, and according to tumor microenvironment expression. We considered as tumor microenvironment the expression of the receptors in nonmelanocytic cells in the dermal infiltrate, such as in macrophages; this was made possible by using a monoclonal antibody against CD163, marker of cells from the monocyte/macrophage lineage.

TAM receptors were assayed using immunohistochemistry. Standard formalin-fixed, paraffin-embedded 4-μm sections were stained using rabbit monoclonal immunoglobulin G (IgG) against human MerTK (catalog no. ab52968, clone no. Y323, 1:50 dilution; Abcam, UK) and a rabbit polyclonal IgG against human AxlTK (catalog no. 8661S, clone no. C89E7, 1:600 dilution; Cell Signaling Technology). A mouse monoclonal IgG against CD163 (catalog no. 760-4437, clone name MRQ-26; Ventana) was used to identify histiocytes. Horseradish peroxidase-labeled goat anti-rabbit IgG (Zhongshan Golden Bridge Biotechnology, China) was used as secondary antibody.

A widely accepted scoring system for immunochemistry does not exist yet. In this study we used a combinative semiquantitative scoring for both of the TAM receptors analyzed (MerTK and AxlTK), which considers the percentage of positive cells and the intensity of immunohistochemical staining in most of the examined fields.

The percentage of positive cells was staged as follows: 0 (0%–10% of positive cells), 1 (11%–50% of positive cells), and 2 (51%–100% of positive cells). This score was calculated arbitrarily, taking a cue from the immunoreactivity score assessed by other reports [11].

The staining intensity was described using a simple qualitative scoring system: “−” score was given for lack of brown immunoreactivity, “+/−” score for very weak staining, “+” score for weak staining, “++” score for moderate staining, and “+++” score for high staining. After that the results were converted into grades: “−” score was assigned 0, “+/−” was 1, “+” was 2, “++” was 3, and “+++” was 4. The 2 scores were added together to obtain an intermediate score with 6 possible values (0 ÷ 6).

The percentage of positive cells and the staining intensity were assessed for each of the following 3 components of the tumor: tumoral melanocytes of the dermoepidermal junction immediately beneath the row of epidermal basal cells, dermal tumoral melanocytes, and the cells of the tumor microenvironment. Finally, the final score for each patient was obtained summing the 3 intermediate scores with 18 possible values (0 ÷ 18).

In each sample a TAM immunohistochemical reactivity of more than 2 was defined as positive.

### Data Processing and Statistical Methods

All histopathological and clinical data for each patient in the study were collected in an Excel database. Each patient was identified with a progressive number to protect his or her identity. The variables of interest were summarized according to an appropriate statistical description on the basis of their type. The score of the expression of the 2 TAM receptors (MerTK and AxlTK) was evaluated by 1 observer.

Statistical analysis was conducted using Stata 9.1 software (StataCorp, College Station, TX, USA) by another person who had no initial knowledge of the detailed clinicopathological features of the lesions. A Spearman rank correlation coefficient was used to evaluate the correlation between TAM receptors’ staining intensity and the clinicopathological variables of the MM samples (univariate analysis).

Subsequently, assuming that the effects of the predictive variables were constant over time, a multiple logistic regression was performed (multivariate analysis). In all statistical methods, P ≤ 0.05 was considered significant.

## Results

A total of 24 patients were included in the analysis. The main clinicopathological features of the sample are reported in Supplementary Table e1, which shows the number of patients for each clinicopathological variable, stratified according to Breslow depth.

### Clinical and Pathological Features of the Sample

In the study were enrolled 6 patients with malignant melanoma pT1 (pathological stage of tumor 1), 6 patients with malignant melanoma pT2, 6 patients with malignant melanoma pT3, and 6 patients with malignant melanoma pT4.

Seventeen patients were men and 7 were women. Mean age of the patients was 56 years (ranging between 29 and 78 years). Regarding the anatomical site, 13 patients showed involvement of the trunk and 11 had an MM localized to the peripheral sites.

A mitotic rate ≥1/mm^2^ was present in 20 patients, while ulceration was present in 11 patients. TIL, assessed only in MM with vertical growth, were present in 7 patients and absent in 10 samples. Regression phenomenon was observed in 7 patients. Four people had an MM with lymphovascular invasion, while in 6 cases MM was associated with a preexisting nevus (25% of the total).

### Correlation of the Expression of Mer and Axl in the Analyzed Specimens

**Figure 1:**
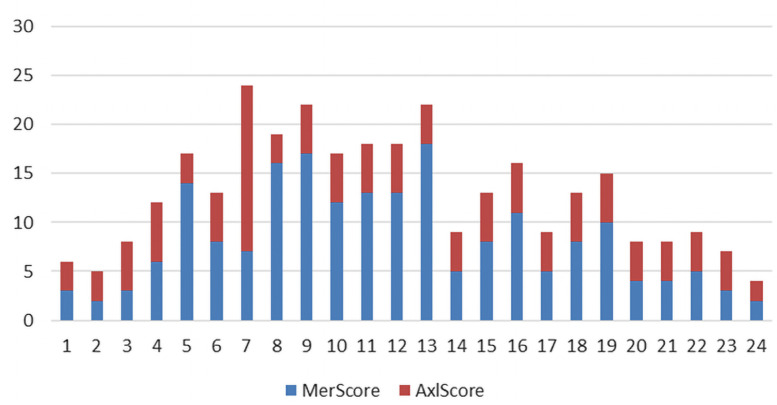
Graphical representation of MerScore and AxlScore for each patient.

Figure 1 shows the trend of MerScore and AxlScore for each patient. The expression of Axl was lower than that of Mer. The main reason for this finding is that MerTK and AxlTK were expressed differently in the MM samples and in the 3 components of the tumor (tumoral melanocytes of the dermoepidermal junction, dermal tumoral melanocytes, tumor microenvironment [Supplementary Tables e2 and e3]).

The cells of the tumor microenvironment contributed to the majority of the total score. The analysis of the CD163 staining revealed that the expression of MerTK was given predominantly by these cells.

MerTK was expressed by tumoral melanocytes and the cells of the microenvironment; in particular, 13 patients expressed MerTK in the dermoepidermal junction’s melanocytes (54% of the patients) and 12 in the dermal melanocytes (50% of the patients). Only 1 patient did not express MerTK in the cells of the tumor microenvironment. In contrast, AxlTK was expressed in all patients by the cells of the tumor microenvironment; only 1 patient exhibited the expression of this TAM receptor also in tumoral melanocytes.

### Expression of MerTK

**Figure 2:**
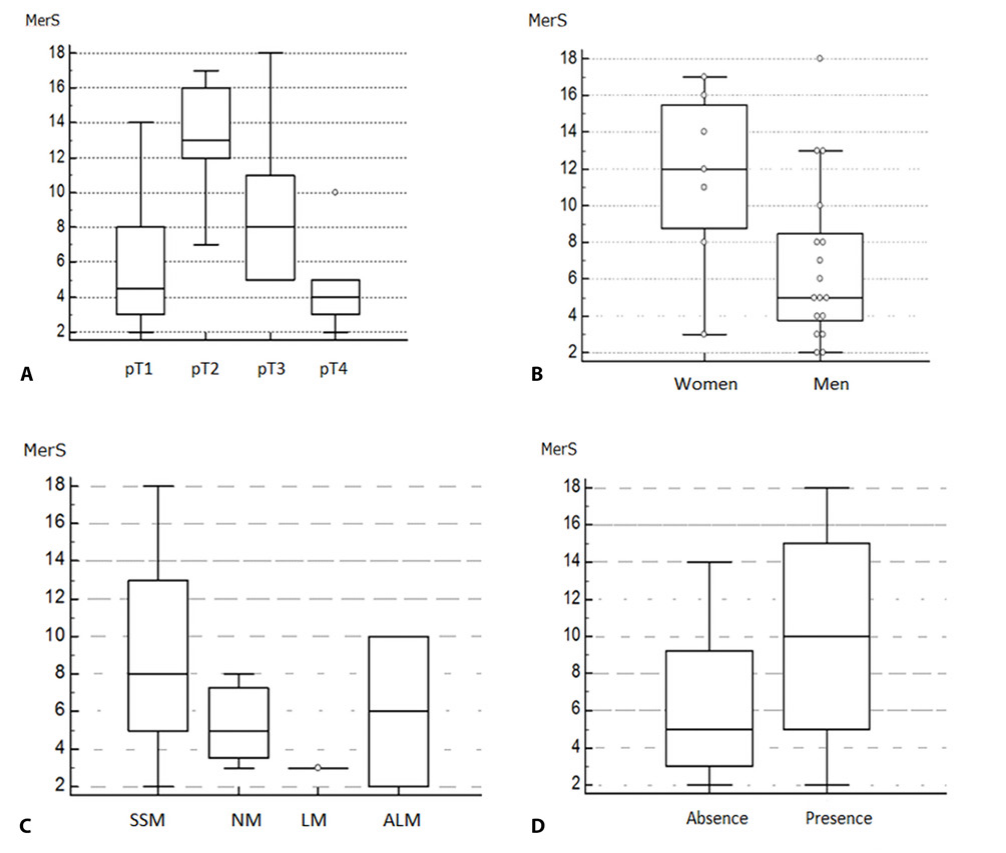
(A) Box plot multiple comparison graph shows the distribution of MerScore (MerS) according to the different stages (pT) of melanoma samples. (B) Box plot multiple comparison graph shows the distribution of MerScore according to sex. (C) Box plot comparison graph shows the variation of MerScore according to the histology of melanoma: superficial spreading melanoma (SSM), nodular melanoma (NM), lentigo maligna (LM), and acral lentiginous melanoma (ALM). (D) Box plot multiple comparison graph shows the distribution of MerScore according to the presence or absence of ulceration.

Evaluating MerScore according to pT stage, we found that mean MerScore was 6 in pT1, 13 in pT2, 9.17 in pT3, and 4.67 in pT4, showing an atypical distribution with a peak in the intermediate stages and lower values in the first and last stages (P = 0.04) (Figure 2A). MerScore was 6.82 in men and 11.57 in women (P = 0.01) (Figure 2B).

The average MerScore was 10.25 in patients ≤60 years old and 6.17 in patients >60 years old (P = 0.06), 7.77 in MMs of the head-neck, thorax-thoracic dorsum, and abdomen, and 8.73 in MMs localized on the limbs (P = 0.8).

Regarding histology, we found that the mean values of MerScore were 9.22 in superficial spreading melanoma, 5.33 in nodular melanoma, 6 in acral lentiginous melanoma, and 3 in lentigo maligna melanoma (P = 0.07) (Figure 2C).

The mean MerScore was 6.6 in patients with a Breslow thickness ≤0.80 mm and 8.63 in those with a thickness ≥0.81 mm (P = 0.4), 4.75 in MMs with mitoses <1/mm^2^, and 8.9 in those with mitoses ≥1/mm^2^ (P = 0.1).

**Figure 3:**
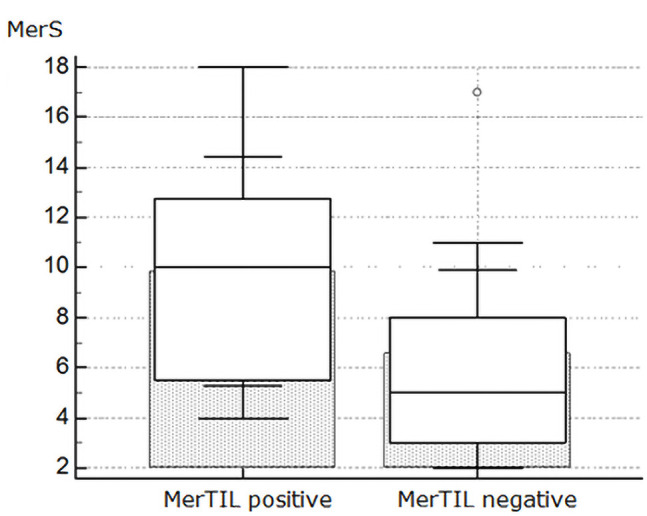
Box plot multiple comparison graph shows the distribution of MerScore according to tumor-infiltrating lymphocytes (TIL).

The average MerScore was 10 in patients with ulceration (P = 0.01) (Figure 2D), 8 in pigmented lesions (P = 0.7), and 6 in lesions with regression (P = 0.1). Mean Mer expression was 9.86 in lesions with TIL and 6.6 in lesions without TIL (P = 0.04) (Figure 3).

The mean MerScore was 11.17 in nevus-associated MMs (P = 0.1) and 4.67 in cases of microscopic satellites (P = 0.1). However, when we performed multiple logistic regression, only the variable ulceration maintained statistical significance (P = 0.03). All these data are summarized in Table 1.

Subsequently, Kaplan-Meier curves were performed for the survival analysis, and the log-rank was used to evaluate the difference between the curves (Supplementary Table e4). According to the expression of MerTK in tumoral melanocytes, the patients were divided into 2 groups: MerTK-negative (MerTK−) lesions and MerTK-positive (MerTK+) lesions. Mean progression-free survival (PFS) was 55.22 months in MerTK− lesions, while it was 93.58 in MerTK+ lesions (P = 0.004); in the long term, we found that overall survival (OS) was 62.9 in MerTK− and 93.76 in MerTK+ lesions (P = 0.02).

### Expression of AxlTK

**Figure 4:**
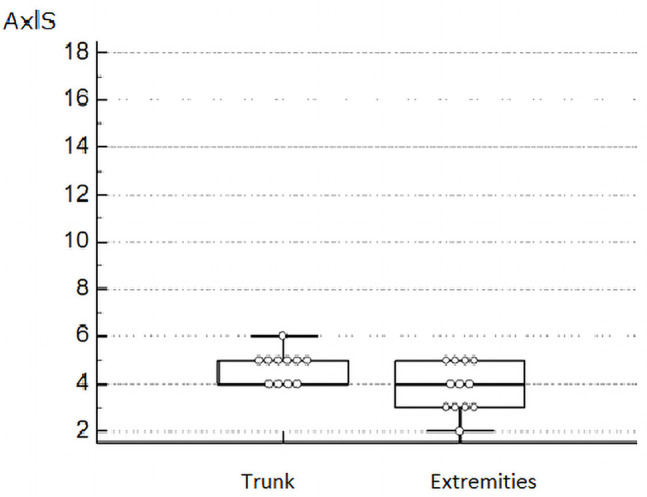
Box plot multiple comparison graph shows the distribution of AxlScore according to the anatomical site (axial: head-neck, trunk; extremities: upper and lower limbs).

The mean AxlScore was 4.17 in pT1, 6.67 in pT2, 4.5 in pT3, and 3.83 in pT4, without reaching statistical significance with a P = 0.4. The statistical significance, in the univariate analysis, was not reached also for the variable sex (5.06 in men and 4.14 in women, P = 0.6). Patients aged ≤60 showed a mean AxlScore of 4.42, while in those aged >60 it was 5.17 (P = 0.5). The lesions localized on the head-neck, thorax-thoracic dorsum, or abdomen, in contrast to those localized to the limbs (arms, legs), showed a mean AxlScore of 5.62 and 3.82, respectively (P = 0.01) (Figure 4).

Regarding histology, we found that mean AxlScore values were 5.11 in superficial spreading melanoma, 4.33 in nodular melanoma, 3 in acral lentiginous melanoma, and 3.5 in lentigo maligna (P = 0.2). The average AxlScore was 4.4 in patients with Breslow thickness ≤0.80 mm and 4.89 in patients with a thickness ≥0.81 mm (P = 0.4), 4.75 in MMs with mitoses <1/mm^2^, and 4.8 in those with mitoses ≥1/mm^2^ (P = 0.3). The average AxlScore was 4.27 in patients with ulceration (P = 0.9), 4.86 in pigmented lesions (P = 0.4), 4.14 in lesions with regression (P = 0.7), 6.75 in lesions with peritumoral vascular invasion (P = 0.5), and 3.83 in patients without perineural invasion. Mean Axl expression was 6.29 in lesions with TIL and 4.1 in lesions without TIL (P = 0.8). The mean AxlScore was 4.33 in nevus-associated MMs (P = 0.9) and 3.67 in the lesions with microscopic satellites (P = 0.3). In the multiple logistic regression only 2 variables maintained statistical significance: anatomical site (P = 0.01) and ulceration (P = 0.03). All these data are summarized in Table 2.

According to the expression of AxlTK in tumoral melanocytes, the patients were divided into 2 groups: AxlTK-negative (AxlTK−) lesions and AxlTK-positive (AxlTK+) lesions. The mean PFS was 76 months in AxlTK− MMs and 94 months in the positive ones (P = 0.5), while the mean OS was 79 months in AxlTK− lesions and 94 months in the positive ones (P = 0.5). In any case, statistical significance was not reached, also due to the low number of AxlTK+ lesions (n = 1) (Supplementary Table e5).

### Subanalysis of Mer and Axl Expression in Microenvironment

Evaluating the expression of Mer according to the microenvironment, we found that the expression was 4 in median Breslow thickness of 2.2 mm (0.24–3.8), 3 in median Breslow of 2.3 mm (0.49–4.1), 2 in median Breslow of 2.4 mm (0.2–4.2), 1 in median Breslow of 2.4 mm (0.9–4.1), and 0 in median Breslow of 0.38 mm. The results did not reach statistical significance (Spearman test) owing to the low number of patients and the high presence of variables (P = 0.7). Performing the same analysis in Axl we found that, regarding the microenvironment, the expression was 4 in median Breslow of 0.2 mm (only 1 case), 3 in median Breslow of 1.17 mm (0.24–4.2), 2 in median Breslow of 2.7 mm (0.38–7), 1 in median Breslow of 4.02 mm (only 1 case). Also in this case significance was not reached (P = 0.1).

## Discussion

MerTK and AxlTK receptors could play important roles in the development of MM: they act as direct drivers of the tumor progression and as inhibitory receptors in the cells of the tumor microenvironment that suppress host immunity. The few published scientific papers that have evaluated the expression of MerTK and AxlTK in MM have been mainly conducted in vitro on samples of MM cell lines [7,9,11]. This study, on the contrary, assessed the expression of these tyrosine kinase receptors with immunohistochemical stains on biopsy of cutaneous MM specimens.

**Figure 5:**
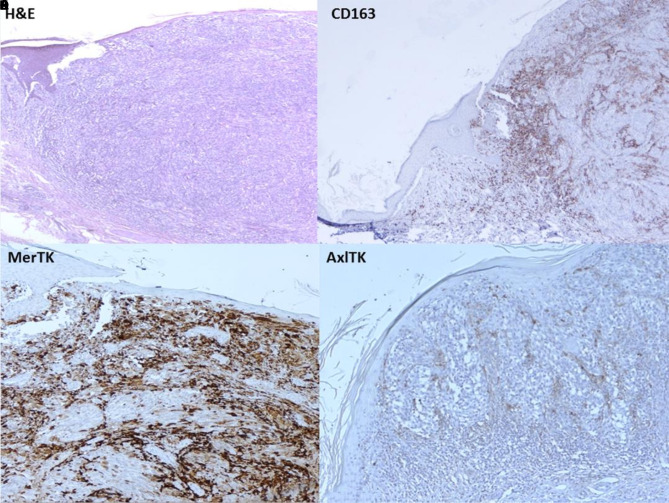
(A) Cutaneous melanoma, pT3 (H&E, ×40). (B) CD163, in the same melanoma as depicted in panel A (×40). (C) MerTK, in the melanoma depicted in panel A (×40). (D) AxlTK, in the melanoma depicted in panel A (×40).

As previously demonstrated [9], we confirmed the expression of MerTK and AxlTK in MM samples with a prevalence of the first receptor (Figure 5, A–D). Our report also demonstrated that these 2 receptors are more expressed by the cells of the tumor microenvironment, basically by tumor macrophages, as also reported in a recent paper [12]. However, contrary to Salmi et al [12], who evaluated mainly the expression of TAM receptors in CD68+ and CD163+ macrophages in benign and malignant melanocytic lesions, our study focused on the correlation between the expression of TAM receptors and the histological main types of MM, with the relative clinicopathological correlations.

We found a significant expression of MerTK in pT2 and pT3 MM, with low levels in initial and advanced tumors; MerTK surely has an important implication in the biological regulation of these 2 stages, and probably this is associated with different biological pathways between the initial tumorigenesis and more advanced primary tumors.

The univariate correlation has also evidenced a significant association between MerTK expression and sex: women have a more elevated expression of this receptor than men (P = 0.04). It is possible to assume a plausible role of the estrogen receptor in determining this difference between the sexes, since data have increasingly demonstrated a role of this receptor in the biology of MM [13,14]. Moreover, it has been demonstrated that breast cancer, a hormone-sensitive tumor, expresses a high level of TAM receptor [15].

The ulceration of the primary tumor also correlates with a more elevated expression of MerTK (P = 0.01). More than half of the ulcerated MMs (6/11) are pT2 and pT3 tumors, which are the stages with discrete proliferation rates and invasiveness. MerTK induces upregulation of focal adhesion kinases, which promotes migration [16]. Moreover, it has been found that MerScore was higher in patients with the presence of TIL (P = 0.04).

The statistical analysis found an association between MerScore of tumoral melanocytes and PFS/OS (P = 0.004 and 0.02, respectively): the patients whose MerTK expression in tumoral melanocytes is higher have a better prognosis; this evidence can be explained by the fact that the presence of TIL correlates with a favorable course [17]. This finding may have predictive clinical implications and could have a role in the organization of the follow-up of the patient with MM; for example, medical examinations could be closer in patients with a low tumoral melanocyte expression of MerTK.

The univariate statistical analysis regarding the expression of AxlTK revealed a significant association between the AxlScore and the anatomical site of the tumor (P = 0.01); in particular, those MMs that are localized to head-neck, thorax-thoracic dorsum, and abdomen have a higher AxlScore than those on arms and legs. This reflects a different importance of this receptor in the MM pathogenesis according to the anatomical sites. It is well known that various genetic alterations differ with the anatomical site of MM, and probably this happens also for AxlTK expression [18].

The main limitation of our study is the small size of the sample. All the correlations between the expression of MerTK and AxlTK with the clinical and histological variables of MM should be validated in a large group of people in order to increase the validity and the impact of the observations. Therefore, a multivariate analysis and a proper sample size could demonstrate the actual role of these TK receptors. However, at the same time, the present findings could also be a consequence of the bias if the receptors show a different pattern of expression according to stage and other prognostic factors.

Another limitation of this study is that the cross-sectional analysis between the TAM receptor scores (AxlScore and MerScore) and the clinicopathological variables of the tumor was done only at the time of its diagnosis. Our analysis established a simple association between these scores and the different variables. An analysis of the expression of these receptors could also be evaluated during the patient’s follow-up, according to the tumor progression in order to hypothesize a pathogenetic correlation.

## Conclusions

This is a preliminary and exploratory study that correlates the clinicopathological characteristics of primary cutaneous MM with the levels of the immunohistochemical expression of MerTK and AxlTK. Moreover, it is a first attempt to evaluate a possible association between the expression of these 2 receptors and the prognosis of the patients. This study could be expanded with the evaluation of the expression of the TAM receptor in metastatic melanomas and the screening of the mutation of the oncogenes nRAS, BRAF, and c-KIT with subsequently therapeutic implications in the era of the “targeted therapy.”

## Supplementary Information

**Supplementary Table e1:** Clinicopathological Baselines of the Sample

	pT1	pT2	pT3	pT4
Sex				
Male	3	3	5	6
Female	3	3	1	0
Age				
≤60 years	3	4	2	3
>60 years	3	2	4	3
Anatomical site				
Head-neck	0	0	1	1
Thorax-thoracic dorsum	2	3	1	2
Abdomen	0	2	1	0
Arms	2	0	0	2
Legs	2	1	3	1
Histology				
SSM	5	6	5	2
NM	0	0	1	2
LMM	1	0	0	0
ALM	0	0	0	2
Breslow depth (mean± SD, mm)	0.47±0.26	1.50±0.28	2.95±0.63	4.59±1.18
Mitoses				
<1/mm ^2^	4	0	0	0
≥1/mm ^2^	2	6	6	6
Ulceration				
Presence	1	3	3	4
Absence	5	3	3	2
TIL				
Presence	0	3	2	2
Absence	1	1	4	4
Pigmentation				
Presence	6	6	5	5
Absence	0	0	1	1
Regression				
Presence	3	1	0	3
Absence	3	5	6	3
PVI				
Presence	0	1	1	2
Absence	6	5	5	4
PNI				
Presence	0	0	0	0
Absence	6	6	6	6
Nevus-associated melanoma				
Presence	0	4	0	2
Absence	6	2	6	4
Microscopic satellites				
Presence	0	0	1	2
Absence	6	6	5	4

ALM = acral lentiginous melanoma; LMM = lentigo maligna melanoma; NM = nodular melanoma; PNI = perineural invasion; pT = pathological stage of tumor; PVI = peritumoral vascular invasion; SD = standard deviation; SSM = superficial spreading melanoma; TIL = tumor-infiltrating lymphocytes.

**Supplementary Table e2:** Expression of MerTK According to 3 Different Components of the Tumor

	pT1	pT2	pT3	pT4
MerTK				
Dermoepidermal junction’s tumoral melanocytes	30.56%	30.77%	21.82%	10.71%
Dermal tumoral melanocytes	13.89%	30.77%	23.64%	10.71%
Tumor microenvironment	55.55%	38.46%	54.54%	78.58%

**Supplementary Table e3:** Expression of AxlTK According to 3 Different Components of the Tumor

	pT1	pT2	pT3	pT4
AxlTK				
Dermoepidermal junction’s melanocytes	0%	15%	0%	0%
Dermal melanocytes	0%	15%	0%	0%
Tumor microenvironment	25%	70%	100%	100%

**Supplementary Table e4:** Survival Analysis According to Mer Expression

	PFS (Months±SD)	P ^a^	OS (Months±SD)	P ^a^
Mer−	55.22±36.3	*0.004*	62.9±35.4	*0.02*
Mer+	93.58±13.6		93.76±11.6	

a Kaplan-Meier product and log-rank test.

Significant values are given in *italic*.

OS = overall survival; PFS = progression-free survival; SD = standard deviation.

**Supplementary Table e5:** Survival Analysis According to Axl Expression

	PFS (Months±SD)	P ^a^	OS (Months±SD)	P ^a^
Axl−	76±31.2	0.5	79±29.6	0.5
Axl+	94		94	

a Kaplan-Meier product and log-rank test.

OS = overall survival; PFS = progression-free survival; SD = standard deviation.

## Figures and Tables

**Table 1:** Analysis of Clinicopathological Variables According to MerTK Expression

	MerScore (Mean±SD)	P^a^	P^b^
Sex		*0.04*	NS
Male	6.82±4.4		
Female	11.57±4.8		
Age		0.06	NS
≤60 years	10.25±4.6		
>60 years	6.17±4.6		
Anatomical site		0.8	NS
Head-neck/thorax-thoracic dorsum/abdomen	7.77±4.5		
Arms/legs	8.73±5.7		
Histology		0.07	NS
SSM	9.22±5.1		
NM	5.33±2.5		
LMM	3		
ALM	6±5.7		
pT		*0.04*	NS
1	6±4.5		
2	13±3.5		
3	9.17±4.9		
4	4.67±2.8		
Breslow depth		0.4	NS
≤0.80 mm	6.6±4.8		
>0.81 mm	8.63±5.1		
Mitoses		0.1	NS
<1/mm^2^	4.75±2.8		
≥1/mm^2^	8.9±5.1		
Ulceration		*0.01*	*0.03*
Presence	10±5.5		
Absence	6.69±4.1		
TIL		*0.04*	NS
Presence	9.86±4.9		
Absence	6.6±4.6		
Pigmentation		0.7	NS
Presence	8±4.6		
Absence	10.5±10.6		
Regression		0.1	NS
Presence	6±4.4		
Absence	9.12±5.0		
PVI		0.1	NS
Presence	4.5±2.1		
Absence	8.95±5.1		
Nevus-associated melanoma		0.1	NS
Presence	11.17±5.5		
Absence	7.22±4.5		
Microscopic satellites		0.1	NS
Presence	4.67±3.1		
Absence	8.71±5.0		

a Spearman test between the variables and MerTK expression.

b Multiple logistic regression between the variables and MerTK expression.

Significant values are given in *italic*.

ALM = acral lentiginous melanoma; LMM = lentigo maligna melanoma; NM = nodular melanoma; NS = not significant; PVI = peritumoral vascular invasion; SD = standard deviation; SSM = superficial spreading melanoma; TIL = tumor-infiltrating lymphocytes.

**Table 2:** Analysis of Clinicopathological Variables According to AxlTK Expression

	AxlScore (Mean±SD)	P^a^	P^b^
Sex		0.6	NS
Male	5.06±3.2		
Female	4.14±1.1		
Age		0.5	NS
≤60 years	4.42±1.2		
>60 years	5.17±3.8		
Anatomical site		*0.01*	*0.01*
Head-neck/thorax-thoracic dorsum/abdomen	5.62±3.5		
Arms/legs	3.82±1.1		
Histology		0.2	NS
SSM	5.11±3.1		
NM	4.33±0.6		
LMM	3		
ALM	3.5±2.1		
pT		0.4	NS
1	4.17±2.9		
2	6.67±4.2		
3	4.50±3.1		
4	3.83±2.1		
Breslow depth		0.4	NS
≤0.80 mm	4.4±1.3		
>0.81 mm	4.89±3.0		
Mitoses		0.3	NS
<1/mm2	4.75±1.3		
≥1/mm^2^	4.8±3.0		
Ulceration		0.9	*0.03*
Presence	4.27±1.00		
Absence	5.23±3.6		
TIL		0.8	NS
Presence	6.29±4.7		
Absence	4.1±1.0		
Pigmentation		0.4	NS
Presence	4.86±2.9		
Absence	4±0		
Regression		0.7	NS
Presence	4.14±1.2		
Absence	5.06±3.2		
PVI		0.5	NS
Presence	6.75±6.9		
Absence	4.4±0.9		
Nevus-associated melanoma		0.9	NS
Presence	4.33±0.8		
Absence	4.94±3.2		
Microscopic satellites		0.3	NS
Presence	3.67±1.5		
Absence	4.95±2.9		

a Spearman test between the variables and AxlTK expression.

b Multiple logistic regression between the variables and AxlTK expression.

Significant values are given in *italic*.

ALM = acral lentiginous melanoma; LMM = lentigo maligna melanoma; NM = nodular melanoma; NS = not significant; PVI = peritumoral vascular invasion; SD = standard deviation; SSM = superficial spreading melanoma; TIL = tumor-infiltrating lymphocytes.
